# Supplementary material for: Are carbon emissions trading and green financial instruments synergistic? -Comprehensive quantitative research based on content analysis
Source: PLoS One. 2024 Mar 7;19(3):e0298601. doi: 10.1371/journal.pone.0298601 (PMC10919601; doi:10.1371/journal.pone.0298601)
Supplement: S1 Data — (ZIP) [file pone.0298601.s001.zip › Data/地级市Z_F工作报告.zip!/╣≤╓▌/░▓╦│╩╨/└┤╫╘╣1⁄2╓┌║┼í╛┬φ┐╦╩2╛▌═°í┐_2004.docx]

各位代表：

现在，我代表市人民政府向大会报告工作，请予审议，并请各位政协委员提出意见。

2003年政府工作回顾

过去的一年，在省委、省政府和市委的正确领导下，在市人大、市政协的监督、支持和帮助下，市人民政府坚持以邓小平理论和“三个代表”重要思想为指导，全面贯彻落实党的十六大、十六届三中全会，省委九届三次、四次全会和市委一届五次、六次全会精神，紧紧依靠全市各族干部群众，克服非典影响，抢抓机遇，加快发展，团结拼搏，艰苦创业，较好地完成了年初确定的目标任务，国民经济呈现良好的发展态势，社会各项事业取得新的成绩。

据初步核算，全市GDP 78.75亿元，同比增长10.4%，撤地设市以来首次实现了两位数增长。其中第一产业实现增加值20.03亿元，同比增长13.0%；第二产业实现增加值33.15亿元，同比增长10.0%；第三产业实现增加值25.57亿元，同比增长8.8%；三次产业构成为25.4:42.1:32.5。财政总收入10.05亿元，同比增长14.6%，提前两年实现财政总收入上十亿元台阶目标。其中地方财政收入5.32亿元，同比增长14.5%。农民人均纯收入1512元，同比实际增长6.62%；城镇居民人均可支配收入6272元，同比实际增长22.8%。

基础设施不断完善。各项重点工程建设取得新进展。清镇至镇宁高速公路建设进展顺利，镇宁至胜境关高速公路开工建设。关岭至兴仁、黄果树至水城高等级公路建成通车；平坝至长顺、普定至镇宁、平坝至引子渡、紫云至黄果树和镇宁至坝草的公路改造基本完工。安顺黄果树机场各项工作正抓紧推进。500千伏输变电工程和换流站工程顺利完工，安顺电厂二期两台机组、平坝引子渡电站三台机组建成并网发电，全市新增装机容量96万千瓦，光照电站等北盘江水电梯级开发前期工作顺利进行。继续实施农网建设与改造项目。“三小”水利工程、“渴望工程”、王二河水库、安西灌区、病险水库除险加固等水利工程进展顺利，黔中水利枢纽工程前期工作进度加快。完成造林任务48.8万亩，其中退耕还林34万亩。建沼气池3000口。完成“坡改梯”4.04万亩。共争取到国债投资项目71个，获得中央预算内专项资金2.94亿元，同比增长40.1%。全社会固定资产投资21.90亿元，同比增长3.8%。“十件实事”总体完成较好。

城市建设步伐加快。城市建设和发展基础性工作得到加强，新一轮城市建设初见成效。编制完成了《安顺市古城历史街区保护规划》、《安顺市城市近期建设规划》、《双阳新城控制性详细规划》、《安顺市消防规划》和《安顺市教育布点规划》等一系列规划,完成了各县县域城镇体系规划、总体规划修编和近期建设规划等工作，市县级详规覆盖率分别达75.0%和48.6%。南华路和塔山东西路改造工程完工，首次实现供电电缆入地，人行道实现无障碍设计并全部铺设具有地方特色的青石板。运输路改造、安顺大桥建设和贯城河治理一期工程按期完工。塔山公园拆迁和龙泉路建设工程全面启动，文庙环境整治和中华东西路拆迁前期工作基本完成。文庙、武庙等文物古迹的保护和恢复工作有新进展。开工建设市区东关经济适用住房5万平方米和垃圾卫生填埋场工程。安顺开发区、黄果树新城和西秀新区建设步伐加快。城市面貌正在发生新的变化，中心城市的辐射带动功能进一步加强。

农村经济健康发展。全年粮食生产实现恢复性增长，粮食总产量达73.86万吨，同比增长41.4%。种植业结构从以粮食生产为主继续调整为粮食作物、经济作物和饲料作物全面发展。品种不断调优、调特，种类向多样化、优质化、精细化和无公害方向发展，为农民增收拓宽了渠道。蔬菜、茶叶、香料、烤烟等经济作物产量、质量不断提高。把发展畜牧业作为产业结构调整的突破口，制定了《关于建设生态畜牧业强市的决定》，完善了相关扶持政策措施。加大对良繁体系的投入，新建市县两级扩繁场8个。引进纯种大约克母猪1500头，灰鹅种群扩繁增加2万只，新增杂交牛7000头，林下养鸡60万羽。畜牧业产值达9.91亿元，同比增长4.4%，占农户家庭收入32%以上，在农村经济中起着重要的支撑作用。农业产业化建设有新的进展。幺铺肉牛交易屠宰市场被列为农业部定点畜禽综合交易批发市场。省级重点龙头企业竞争力不断增强，安顺同鑫公司被新列为省级重点龙头企业。龙头企业带动了产业化经营，促进了产业化建设，提高了农牧产品附加值。乡镇企业完成增加值21.67亿元，同比增长12.0%。

扶贫开发力度加大。认真落实《关于加大新阶段扶贫开发工作力度的实施意见》，进一步完善了扶贫开发工作机制。对市直90个单位确定了挂钩扶贫联系点，市县两级选派800余名干部到6个一类重点乡镇、400个一类重点村任职进行帮扶，为新阶段扶贫开发奠定了良好的工作基础。抓好扶贫项目管理，搞好扶贫项目申报，管好用好各类扶贫资金。推广农业适用技术，加大对贫困地区干部和农民的培训力度。有4.7万农村贫困人口基本解决温饱。实现农村极贫人口易地扶贫搬迁921户、4428人。完成了400公里通村公路建设。解决了农村6.51万人的饮水困难。减免农业税及附加450万元。实现城乡供电同网同价，减轻农民负担3600万元。

工业经济稳步增长。规模以上工业总产值完成44.44亿元，同比增长20.5%；规模以上工业增加值完成13.51亿元，同比增长19.8%。地方工业企业更新改造完成3.6亿元，同比增长16.2%。支持地方中小企业延长产业链，带动相关行业发展。加强煤炭等重点矿产资源的勘探与开发，支持大中型煤矿建设，全市新增230万吨原煤生产能力，保证了电力生产和群众生活用煤需要。全市售电量25.59亿千瓦时，同比增长28.0%，增幅为历年最高，有力地支撑了地方经济发展。药品生产、经营企业的GMP、GSP改造和发展呈良好态势。加强同军工企业的联系，市域内军工企业以成功试飞新型高级教练机为标志，取得新的发展。非公有制企业实现工业总产值 32.23亿元，同比增长23.3%，占全市工业经济的57.7%，拉动了全市工业的发展，带动了工业经济结构调整。

第三产业加快推进。明确提出旅游兴市的发展战略和“走进自然、走进历史、走进文化”的旅游业发展定位。进一步理顺了黄果树风景名胜区的管理体制。成功申报为省级优先发展重点旅游区。成功举办油菜花旅游节和格凸河“神奇之旅，神秘之旅”首游式。旅游投入进一步加大，旅游基础设施和旅游推介不断加强。对全市景区景点旅游市场秩序和国道、省道以及重点景区公路沿线公（坟）墓进行了集中整治。旅游业接待国内外游客141.1万人次，同比增长11.7%，实现营业收入3.40亿元，同比增长2.4%。全部金融机构存款余额95.94亿元，同比增长12.4%；贷款余额97.69亿元，同比增长6.5%。房地产、餐饮、商贸、交通运输、邮电通信等服务业加快发展。第三产业拉动经济增长2.9个百分点。社会消费品零售总额28.35亿元，同比增长10.9%，居民消费价格总水平比上年上涨0.6%。

发展环境逐步优化。着力改善发展环境，加大招商引资工作力度。开展建设“诚信安顺”、优化发展环境活动，出台了《安顺市投资者投诉管理暂行规定》、《安顺市行政执法责任制暂行规定》、《安顺市行政执法错案责任追究办法》等规范性文件，切实保护投资者的合法权益和非公有制经济发展。开展了“纪律在你身边”、“群众评议机关”等活动，对市级51个单位和部门进行了群众评议，机关作风建设进一步改进。加大城市管理和综合治理力度，实施城区道路交通“畅通工程”，实行“门前三包”责任制和“包保”责任制，城市脏乱差的状况有较大改善，城市管理达到D类城市三等管理水平，上了一个新台阶。整顿和规范市场经济秩序取得新进展，重点开展了对药品、食品、成品油以及毒鼠强的专项整治，严厉打击制假、售假、偷税、逃税等违法犯罪行为。推行“阳光政务”，各级政府部门向社会公开职能职责和办事程序，更新建设安顺市人民政府网站和“黄果树之窗”网站，扩大了政务公开范围和渠道。全市引进区外投资实际到位资金5.27亿元，同比增长95.4%。实现外贸进出口4535万美元，同比增长123.8%。与国家开发银行签订开发性金融合作协议。与青岛市的对口帮扶工作，与友好城市和民间组织的交流与协作进一步加强。

各项改革继续深化。进一步加大国有企业改革改制力度。青松烟厂政策性关闭破产，起重设备厂实现平稳关闭，虹山轴承总公司政策性破产工作正按程序进行；完成顺健制药厂和中药厂转让，实现中药饮片厂和玖久药业的资产重组，市药材公司和利康医药公司整体改制工作顺利完成。财政管理体制改革进一步完善。继续加快建立公共财政步伐，进一步完善乡镇财政体制，加强税收征管，推行部门预算，调整优化支出结构，规范政府采购行为，加强财政性资金管理，强化“收支两条线”。继续扩大和巩固农村税费改革试点工作成果，完善相关政策措施。国有粮食购销企业改革进展顺利，实现了粮食购销市场化。农村信用社改革和农村合作医疗改革试点工作积极推进。支持城市信用社加快发展。行政审批制度改革迈出新步伐，共取消市级行政审批事项143项。实施非经营性政府投资项目代建制，推进建设项目投、建 、管、用的分离。深化住房制度改革，推进房产上市交易。落实了中央调整机关事业单位职工工资和增加离退休人员离退休费的政策。人事制度改革不断深化，公开招考录用国家公务员工作不断完善，启动了事业单位人事制度改革工作。

社会事业协调发展。面对突如其来的非典疫情威胁，始终把人民群众的生命安全放在第一位，建立市县乡三级防治工作领导机制，努力抗击非典，市县两级共投入近1000万元防治经费，确保了全市没有一例非典病例发生。建成安顺市疾控中心、普定县疾控中心、镇宁自治县疾控中心，市中心血站投入使用。推进市县疾病预防控制体系、医疗救治体系、市县乡疫情信息体系建设，卫生执法监督体系得到加强，应对重大灾害和解决各种突发事件的能力得到提高。西秀区“两基”工作通过省评估验收。全市“普九”人口覆盖率提高到61.03%。新建“义教工程”项目学校 27所、“普九”工程项目学校25所，完成“危改工程”项目学校9所。扩大了普通高校和高中招生规模，民族教育、职业技术教育和成人教育得到发展。大力推进科技进步，积极实施科技创新，推动科技成果转化为生产力。实施文化精品战略，《安顺地域风情散文——神秀黔中》、《黄果树之声》等一批作品产生了良好的社会效益。重视广播电视播出安全工作，改造和建设农村卫星广播电视收转站383座。加强计划生育服务与管理，人口出生率和自然增长率分别控制在17.18‰和10.25‰以内，同比下降1.52和1.45个千分点。计划生育率为88.02%，同比上升4.85个百分点。巩固提高“村为主”，稳妥推进“村民自治”。资源和环境保护工作得到加强。

就业再就业和社会保障工作进一步加强。认真贯彻落实中央和省关于促进就业和再就业的工作部署，高度重视就业和再就业工作。市级共拨付资金288.5万元用于再就业工作和“两个确保”。启动下岗失业人员小额担保贷款工作，落实减免税费等优惠政策，维护职工和劳动者合法权益。发放《再就业优惠证》8641本。全年实现就业安置7495人，培训下岗失业人员993人，促进下岗失业人员再就业3151人（其中“4050”人员203人）。积极引导大中专毕业生就业。城镇登记失业率控制在4.5%。劳务输出27297人，劳务输出收入2亿元。为民工追回拖欠工资238万余元。继续巩固“两个确保”100%的成果，切实做好“三条保障线”的衔接。加强对社会保险基金的管理，企业职工基本养老保险制度和失业保险制度不断健全和完善。平稳推进城镇职工基本医疗保险工作。

政治文明和精神文明建设成效显著。坚持依法行政，自觉接受市人大和市政协的监督。人大代表、政协委员的建议和提案回复率保持100%。厂务、村务公开不断深化，基层民主进一步加强。贯彻落实《公民道德建设实施纲要》，着力提高人们的思想道德水平。以“四创”活动为龙头，广泛开展“改陋习、树新风”活动和群众性精神文明创建活动，涌现出一批文明社区、文明行业、文明单位、文明村镇，城乡文明水平不断提高。国防后备力量建设力度加大，“双拥”工作扎实推进，平坝县荣获“全国双拥工作模范县”称号，关岭自治县人武部荣获“全军造林绿化先进单位”称号，军政军民团结更加巩固。深入开展“四五”普法教育，全民法制观念逐步增强。加强社会治安综合治理，严厉打击黑恶势力犯罪团伙和“法轮功”等邪教组织，深入开展专项整治斗争，深入开展“扫黄打非”，扫除社会丑恶现象；加强信访工作，认真接待群众来信来访，及时化解和处理矛盾，认真解决各类遗留问题，积极稳妥地处置群体性事件，维护了社会稳定。严格执行安全生产责任制，大力开展以道路交通、煤矿安全、非煤矿山、危险化学品、公共聚集场所消防安全和公路超载超限运输为重点的专项整治，安全工作得到加强，安全生产形势总体稳定。认真贯彻落实党的民族宗教政策，民族宗教工作不断加强，民族团结，宗教稳定。重视发挥工会、共青团和妇联等人民团体联系广大人民群众的桥梁和纽带作用。妇女儿童、老龄和残疾人事业健康发展。外事、侨务、人防、体育、档案、史志等工作取得新成绩。

各位代表，过去的一年，是我市克服非典影响，经济持续快速健康发展的一年；是改革开放全面推进，物质文明、政治文明、精神文明建设取得明显成效的一年；是城市发展变化较大，人民群众得到较多实惠的一年。这是省委、省政府和市委正确领导的结果，是市人大和市政协监督、支持的结果，是全市人民奋力拼搏的结果。在此，我代表市人民政府，向全市工人、农民、知识分子、各级干部和离退休老同志，向人大代表、政协委员，向各民主党派、工商联、人民团体、无党派人士，向驻安部队、武警官兵、政法公安干警和驻安单位，向所有关心、支持安顺改革开放和现代化建设的各界人士致以崇高的敬意和衷心的感谢！

在充分肯定成绩的同时，我们也要看到经济社会发展中还存在不少困难和问题：城市经济内在活力不强，尚未形成大规模的生产力积聚，辐射带动能力弱；农村基础薄弱，农业产业化经营水平不高，农民增收渠道狭窄，解决农村贫困问题任务还很艰巨；地方国有企业改革改制难度较大，工业经济竞争力不强，企业整体素质不高；固定资产投资新增因素不多，招商引资步子不大；电力供应紧张，重特大安全生产事故时有发生；科技、教育、文化、卫生等社会事业发展较为落后，生态环境脆弱，人口形势依然严峻；就业和社会保障压力大；新形势下的群众工作还要进一步加强，维护社会稳定任务重，等等。这些问题，我们将高度重视，认真对待，采取有力措施，切实加以解决。

2004年政府工作主要任务

今年政府工作的总体要求是：以邓小平理论和“三个代表”重要思想为指导，全面贯彻十六大、十六届三中全会、中央经济工作会议、中纪委三次全会、省委九届四次全会、省经济工作会议、省纪委三次全会、省十届人大二次会议和市委一届七次全会精神，坚持以人为本，牢固确立和认真落实全面、协调、可持续的发展观，按照稳定政策、适度调整，深化改革、扩大开放，把握全局、解决矛盾，统筹兼顾、协调发展的思路，以求真务实的精神，做好各项工作。进一步落实扩大内需的方针，抢抓西部大开发和新阶段扶贫开发的机遇，大力实施科教兴安、开放带动、人才强市和可持续发展战略，加快推进农业产业化、新型工业化和城镇化，保护好、引导好、发挥好各方面加快发展的积极性，切实把工作重点转到调整经济结构、转变增长方式、提高增长质量和效益上来，促进国民经济持续快速协调健康发展和社会全面进步。

国民经济和社会发展的主要预期目标是：（1）GDP增长10%以上，其中第一产业增长6%以上，第二产业增长13%，第三产业增长10%以上。（2）全社会固定资产投资增长12%以上。（3）财政总收入和地方财政收入分别增长10%以上。（4）社会消费品零售总额增长10%。（5）农民人均纯收入和城镇居民人均可支配收入分别增长5%以上。（6）城镇登记失业率控制在4.7%以内。（7）人口出生率和人口自然增长率分别控制在16.5‰、10‰以内。（8）新增5万农村贫困人口越过温饱线。

今年是全面落实党的十六大、十六届三中全会精神，深化改革、扩大开放、促进发展的重要一年；是实现“十五”计划的关键一年；也是我市形成以城市为中心组织区域经济社会发展整体格局的一年。做好今年政府各项工作，意义重大。要按照胡锦涛总书记提出的大力弘扬求真务实精神、大兴求真务实之风的要求，立足我市经济社会发展实际，保持清醒的头脑，增强加快发展的责任感和紧迫感，自加压力，负重奋进，确立农业稳市、旅游兴市、环境立市、工业强市的发展战略思路，并把实施人才战略贯穿其中。围绕市场需求，深化对发挥安顺优势的认识，认真总结安顺多年来持续稳定发展取得的成绩和经验，充分调动和发挥各方面的积极性，保持良好的精神状态，以结构调整为主线，以深化改革为动力，用改革的办法，着力从思想观念上、体制机制上、环境建设上解决阻碍改革开放和经济社会发展的问题，千方百计保持经济较快增长，促进社会全面进步，实现好、维护好、发展好最广大人民群众的根本利益。在具体工作中，着重抓好以下十个方面：

一、深入贯彻落实中央扩大内需的方针，努力发挥投资拉动经济增长的龙头作用

（一）继续抓好西部大开发重点项目建设。配合做好清镇至镇宁、镇宁至胜境关高速公路等骨干公路为重点的交通基础设施建设；配合做好黄桶至织金铁路前期工作。推进光照电站、马马岩和董箐梯级电站、三岔湾电站以及一批小水电建设，进一步做好安顺第二电厂及以黄家湾水库为龙头的蒙江流域水电开发前期工作，实施好县城电网改造工程项目。配合做好黔中水利枢纽工程前期工作。继续实施以退耕还林和荒山造林为重点的生态建设，加快石漠化治理步伐。按照国家和省“十一五”规划编制工作的总体部署，启动全市“十一五”规划编制工作。

采取有力措施，确保全面完成市委、市政府确定的18件实事：1、确保农民人均纯收入增长5%以上。2、解决农村50000贫困人口的温饱问题。完成易地扶贫搬迁1060户、4500人。3、实施“渴望工程”，解决农村11000户、50000人饮水困难。4、建成中华东西路友爱路段。完成龙泉路建设工程和马鞍山路改造工程。5、完成塔山公园和文庙环境整治建设工程。6、开工建设市级人才和劳动力市场。7、城镇新增就业安置11000人，下岗失业人员再就业3900人（其中“4050”人员再就业400人）。8、完成100公里通乡油路工程、1000公里通村公路工程。完成紫云至罗甸公路紫云段油路改造工程。9、开工建设50000平方米经济适用住房。完成第一期廉租房建设。10、在市区新建和改造垃圾中转站4座、公共厕所8座。11、完成王二河水库引水工程。完成屯堡文化旅游区道路基础设施建设项目。举办2004年中国贵州安顺黄果树瀑布节暨蜡染艺术节。12、完成省下达的农村沼气池建设任务。13、初步建成县乡村三级畜禽疫病防治体系。14、建设农村党员远程教育网络点300个。15、建设“义教工程”项目学校7所。改、扩建农村中小学41所。为一类扶贫重点乡镇的18000名中小学生免费提供教科书。确保镇宁自治县、关岭自治县通过省“两基”验收。16、改造和建设农村卫星广播电视收转站200座。17、改、扩建乡镇计生服务站12个。新建村级计生服务室350个。18、建设市级紧急救援中心和西秀区、平坝县、关岭自治县、紫云自治县疾控中心。建设市和5县传染病区。

(二)进一步提升城市品位。充分发挥规划的龙头作用，加快城市基础设施建设，加强城市管理。启动黄果树大道工程建设和贯城河下段河道治理工程，加大“城中村”的改造力度，实施农民新村规划建设；加强房屋拆迁管理，依法规范拆迁行为；加大对违法违章建设行为的查处力度，依法拆除违法违章建筑。完善城市管理体制，在市区范围内尽快开展相对集中行政处罚权工作。强化城区、街道、社区管理城市的职能，把责任落实到基层，继续完善和落实“门前三包”责任制和“包保”责任制，进一步巩固和扩大城市环境综合整治成果，建立城市长效管理机制。支持西秀新区加快发展，积极推进开发区建设成为双阳区，不断壮大中心城区规模，发挥辐射带动作用。

（三）积极推进投资主体多元化。推进投资体制改革，进一步拓宽投资渠道。减少准入限制，落实企业投资自主权，凡法律、法规和政策没有明确限制的领域，对所有投资者全面开放。积极引进优强企业投资我市电力和煤炭项目建设，多渠道筹措项目资本金。对煤炭资源的勘探以及煤矿的设计建设，逐步通过公开招标确定项目法人。加快水利投资体制改革，抓紧研究制定水资源有偿使用的实施办法。通过鼓励农民投工投劳等形式，探索解决水利设施地方配套资金的具体途径和办法。对小型水利工程，要明确所有权、放开建设权、拍卖使用权、搞活经营权。改革市政公共事业管理体制，推进政企分开、政资分开、政事分开，鼓励民营资本、外来资本和混合所有制资本采取多种方式参与城市基础设施建设和政府公共产品经营。加强与国家开发银行授信合作项目资金管理，确保资金专款专用，确保投资效益。

二、切实把解决“三农”问题摆在重要位置，千方百计促进农民增收

（一）高度重视保护和提高粮食综合生产能力。巩固农业基础地位，增强粮食安全意识，确保全年粮食播种面积240万亩，粮食总产量达到75万吨以上。严格执行基本农田保护制度和耕地占补平衡政策，未经批准，严禁在基本农田保护区内进行各种非粮食生产的开发用地。以土地综合整治、改造中低产田土和提高现有“坡改梯”工程质量为重点，加大基本农田建设力度，配套建设“三小”水利设施，增加有效灌溉面积，完善排灌设施，提高水资源利用率。充分发挥样板的示范带动作用，进一步优化品种结构，选择推广一批高产优质良种和先进耕作配套技术，增强抗御自然灾害的能力，进一步提高优质粮食作物比重，提高单产，增加总产。加强农业科技推广体系建设，健全乡镇农技推广网络，搞好农业适用技术培训，大力推广农业适用技术，提高技术入户率。认真抓好粮食收购和储备工作，不断提高政府对粮食市场的调控能力。

（二）加大农业结构调整力度。以专业村为基础，以协会为载体，继续推进“一乡一特、一村一品”，扩展“多乡一特、多村一品”。围绕建设生态畜牧业强市的目标，认真贯彻落实《关于建设生态畜牧业强市的决定》，继续把畜牧养殖业作为农业结构调整的突破口，加快发展生猪生产，加快发展肉牛、肉禽和地方名优品种畜禽，努力提高畜牧渔业产值在农业总产值中的比重。加快良种繁育体系和防疫体系建设，突出抓好高致病性禽流感等畜禽疫病防治工作。以提高优质农产品和特色经济作物比重为重点，实施优势农产品区域布局规划，大力发展无公害食品。积极调整林业结构，以坑木林、优质经果林、森林生态旅游业为重点，发展壮大林业生产，着力解决退耕农户的长远生计问题，处理好保护与发展的关系。

大力推进农业产业化经营。加大对农产品生产、加工和运销龙头企业的扶持力度，鼓励和支持各类市场主体从事农业产业化经营，通过股份制、股份合作制等方式与各类农村合作经济组织及广大农户建立有效的利益联结机制，形成利益共同体。积极引进市外优强企业参与我市农业产业化经营，不断提高农产品的附加值和市场竞争力。大力发展农产品加工、保鲜、贮藏、运输等行业，发展订单农业。认真做好农业部定点的断桥农产品批发交易市场和幺铺畜禽综合交易批发市场的建设工作。充分发挥贵黄公路沿线及城郊农业的示范带动作用，整合技术、市场、信息、资金等资源，推进农业示范基地建设。

（三）引导农村富余劳动力有序转移。把农村富余劳动力向非农产业和城镇转移纳入经济社会发展规划，作为增加农民收入的重要渠道。建立健全各级劳务输出管理机构，加强农村劳务输出组织协调和信息服务等方面的工作，提高农民的组织化程度。增加对农民工培训的投入，安排一定的资金用于农村劳动力技能培训，同时要多渠道筹集农民工培训资金，逐步形成政府扶持、培训机构减免收费、农民适当分担的投入机制，鼓励中介机构及用人单位对农民工进行定单培训。切实维护农民工的合法权益。继续清理歧视农民工的政策规定和各种乱收费，在劳动保护、户籍管理、子女就学等方面给予农民工平等待遇。着力解决建设领域拖欠工程款以及拖欠农民工工资问题。

（四）加快新阶段扶贫开发工作。把扶贫开发作为事关全局的重大问题，继续贯彻落实《关于加大新阶段扶贫开发工作力度的实施意见》，做好新阶段扶贫开发工作。认真落实全部免除一类扶贫重点乡镇贫困人口负担的农业税及附加的政策；认真落实免除一类扶贫重点乡镇贫困户儿童的杂费和书本费政策。扎实做好6个一类重点乡镇、400个一类村的扶贫帮扶工作。在两年内完成对一类扶贫重点乡镇农村中小学所有D级危房的改造。按照统一部署、有序推进、落实到村的要求，加强农村水、电、路、通信等基础设施建设，着力改善生存生产环境，增加农民收入。健全扶贫投入机制，加强资金管理，提高使用效益。所有扶贫资金的使用，都要实行公示、公告和报账制度，严格监督和审计，真正使贫困户受益。注重发挥民兵预备役成建制队伍在新阶段扶贫开发中的作用。

三、发挥地方优势，推进产业结构优化升级

（一）加快发展能源工业。抓住“西电东送”的有利时机，促进我市能源工业实现跨越式发展。创新体制和机制，大力发展电力工业，采取合作办电、入股办电、出让经营权等方式，吸引各方面的资金、技术共同参与电力开发。加强对煤炭资源的普查和详查，在西秀区、平坝、普定、镇宁、关岭等地发展一批大中型煤矿和电煤基地；积极发展煤化工，提高煤资源的综合利用水平；加强煤炭“五统一”税费征收管理。制定和完善电力工业上下游产业发展规划，建立电力与相关产业配套发展的利益联接机制，运用经济杠杆，缓解电力对经济发展的“瓶颈”制约作用,协调推进相关产业发展。

（二）稳健发展高耗能产业。在搞好环境保护、严格控制污染、遵守国家产业政策的前提下，搞好高耗能产业的发展。按照科学规划、合理布局、提高产业集中度的要求，加大对优强企业的扶持，鼓励市内外企业通过参股、控股、收购等方式，参与铝、铁合金、水泥等高耗能企业的整合与重组，提高经济效益和市场竞争力。同时，要加强宏观调控，贯彻遏制高耗能企业盲目扩大投资和低水平重复建设的政策措施，避免造成环境污染和资源浪费，促进高耗能产业健康发展。

（三）壮大地方优势产业。大力发展制药业，支持制药企业通过股权置换、合并重组等方式进一步提升实力，拓展市场，扩大规模。支持全市药品生产、经营企业在规定的时间内通过GMP、GSP认证。支持百灵制药等药业企业做强做大。搞好地方民族药单方、验方的挖掘和保护，开发拥有自主知识产权的新特药。积极引导制药企业建设集中连片、规范的中药材种植基地。鼓励发展牛肉、山药、辣椒等农副产品精深加工项目，支持“牛来香”、华泰、安顺酱菜厂、安顺油脂化工厂等农副产品加工企业进一步壮大实力，加快发展，形成一批有一定规模和影响的绿色食品、无公害食品加工企业，拓宽轻工业发展领域。大力发展烤烟生产，用足用好计划资源，优化种植布局，增加科技含量，提高优质烟叶比重，增强烟叶竞争力，建立烤烟种植风险保障体系，切实保护烟农的生产积极性，维护烟农利益。完善扶持高新技术产业发展的政策措施，加快推进辣椒素晶体等高新技术产业化。有计划、有步骤地振兴蜡染等地方传统名优产品。

（四）积极支持军工企业发展。加强与军工企业的联系，积极为军工企业协调解决各种实际问题，为军工企业的发展创造环境，搞好服务。支持军工企业在发展以航天航空为重点的高新技术产业的同时，提高汽车、机械、精密仪器等制造业发展水平。支持军工企业的技术改造，发挥军工企业人才、技术、设备、品牌等综合优势，加快军转民步伐，带动我市制造业水平的提高，使军工企业成为我市新的经济增长点。引导和支持云雀轿车等民品生产项目与优强企业联合，促进产品档次和生产规模上台阶。

四、大力发展非公有制经济，不断增强经济增长的活力

（一）扩大多种经济成份共生繁荣改革试验。认真总结和推广多种经济成份共生繁荣改革试验区经验，积极探索，大胆实践，勇于创新，进一步深化、拓展改革试验，把多种经济成份共生繁荣改革试验区扩大到全市。继续清理限制非公有制经济发展的相关政策规定，制定和落实保护非公有制企业发展的具体办法，把加快发展非公有制经济纳入全市经济社会发展计划，纳入目标管理体系。完善商务中心“一站式”办公，建立全方位、多层次的服务支持体系。改革政府对非公有制企业的审批管理，对一些项目及事项逐步改审批制为备案登记制。要强化政策保障、组织保障、法制保障，为非公有制经济发展创造宽松开明的政策环境、高效优质的服务环境、规范公正的法治环境、稳定有序的社会环境、洁净文明的人居环境。尊重和保护非公有制企业合法权益。切实解决非公有制经济多头管理和无人管理的问题。

（二）大力发展非公有制经济。拓宽非公有制经济的进入领域，凡是国家法律、法规未明确禁入的领域，非公有制经济都可以进入。鼓励和支持非公有制经济参与基础设施建设和生态环境建设，并通过参股、控股、租赁、兼并、联营等方式，参与国有企业改革；引导非公有制经济发展农副产品加工、储存运输和特色产业；引导非公有制经济从主要集中在第三产业向一、二产业延伸；引导非公有制企业提高素质，依法经营，照章纳税，保障职工合法权益。扶持一批产值大、税收高、市场竞争力强的非公有制骨干企业进一步壮大规模，提高产业关联度，增强带动能力。

五、实施旅游兴市战略，加快第三产业发展

（一）努力建设最佳旅游目的地。认真贯彻落实省委《关于加快旅游业发展的意见》，抓住省对优先发展重点旅游区加大支持的机遇，树立大旅游观念，充分发挥我市旅游资源品位高、组合好、景区可进入条件好的优势，以游客在安顺停留3天以上为目标，整合全市旅游资源，促进旅游业实现新的突破。扶持重点旅游景区优先发展，发挥国家级风景名胜区、国家地质公园和屯堡文化的品牌效应，推出旅游精品，提高旅游产品档次和水平。加强旅游基础设施建设，重点抓好黄果树新城建设。以黄果树为龙头，优化旅游线路设计，强化宣传推介，健全客源开发机制，加强对市内旅行社的扶持和培育，加强与省外旅行社的合作，搞好以长三角、珠三角为重点，面向全国，辐射东南亚，辅之以欧美的客源市场的培育与开发。推广天龙屯堡旅游发展模式，拓宽旅游投资渠道，探索社会化参与、多元化投资、市场化运作的方式，选择部份景区、景点进行特许经营试点。开发乡村旅游，发展旅游扶贫。依法保护民族民间文化和自然遗产。继续做好格凸河国家级风景名胜区的申报工作。办好屯堡文化节。

（二）加快第三产业发展。创造良好的政策和体制环境，推动各类服务业加快发展。优化创业环境，鼓励和支持社会力量投资兴办各类服务业。对商业流通、交通运输、饮食服务等传统服务业通过实现充分竞争，加快发展。对会计、咨询、房地产、信息、法律服务等新型服务业通过规范市场秩序，促进健康发展。大力发展现代物流业，鼓励流通行业的联合与重组，推进流通领域信息化，运用现代流通方式整合市场资源，提高商品市场的组织化程度。

六、加快改革开放步伐，为经济社会发展注入新的动力

（一）抓好国有企业改革。以建立归属清晰、权责明确、保护严格、流转顺畅的现代产权制度为突破口，推进以股份制为主的公有制经济的多种实现形式，大力发展国有资本、集体资本和非公有制资本等参股的混合所有制经济，实现投资主体多元化。加大国有企业产权转让工作力度，采取股份制改造、出售、公开拍卖、重组等多种形式，在今、明两年内，市县（区）属国有企业百分之百改制，职工身份百分之百转换。在国有企业改制重组过程中，杜绝国有资产流失。落实困难企业关闭破产和兼并重组的各项政策，加快国有困难企业退出市场步伐，妥善解决资产处置、银行债务、人员安置等问题，着力盘活优良资产，实现体制和机制的创新，使企业走上新的发展道路。抓好虹山轴承总公司破产重组和利达公司改制工作。按照政企分开的原则，建立起管人、管事、管资产相结合的制度，加强国有资产的监督管理，确保国有资产保值增值。

（二）抓好农村经济改革。巩固和发展农村税费改革成果，落实今年降低农业税税率1个百分点和取消烤烟以外的农业特产税的政策，完善对种粮农民进行直接补贴的办法，逐步建立健全新税费制度下有效的农民负担监督管理机制。继续深化粮食流通体制改革。保障农民对土地承包的合法权益。改革土地征用制度，给予农民合理的征地补偿，妥善安置失地农民。按照依法、自愿、有偿的原则，深化土地流转制度改革，培育土地使用权市场，促进土地资源向土地资本转变。按照“从严控制，先行试点，逐步发展”的原则，抓好农村信用社改革试点工作。扩大新型农村合作医疗试点范围，建立完善农村特困医疗救助制度、对口支援农村卫生工作制度和农村巡回医疗制度。

（三）深入推进其它各项改革。深化行政审批制度改革，继续清理和取消不符合规定的行政审批事项。继续深化财税体制改革，切实加强增收节支，积极推行国库集中收付制度，进一步完善部门预算，加强政府采购管理，严格实行“收支两条线”，提高财政资金使用效率。全面推行非经营性政府投资项目代建制。加快发展地方性金融机构，规范发展城市信用社，推进城市信用社建成商业银行。进一步深化住房分配货币化改革。稳妥推进事业单位人员聘用制改革。

（四）不断扩大对外开放。着力建设“诚信安顺”、优化发展环境，形成运转高效的工作机制和对内开放与对外开放相协调的管理体制，严格保护各类投资者的合法权益，严肃追究损害投资环境的行为，积极为外来投资者解决各种实际问题。探索和创新招商引资方式，实行项目招商、产业招商、优势资源招商，加大优势资源、优质资产的开放力度，以开放促进开发，建立招商引资政府奖励制度，提高招商引资实效。以资源开发为纽带，以共同发展为目的，加强与周边市州地的优势互补和区域合作。加强与青岛的对口帮扶工作，搞好与友好城市的协作。

七、关心人民群众生产生活，抓好就业再就业和社会保障工作

（一）抓好就业和再就业工作。坚持劳动者自主择业、市场调节就业和政府促进就业的方针，加强领导，统筹安排，认真落实促进就业和再就业的各项政策措施。进一步强化政府公共就业服务功能，加强市场体系建设，发展劳动力、产权、资本、技术等要素市场，不断完善劳动力市场功能。把经济结构调整与扩大就业有机结合起来，注重发展劳动密集型产业，注重扶持中小企业，注重发展非公有制企业，通过发展创造更多的就业机会，不断提高经济增长对就业的吸纳能力。强化目标责任制管理，加快《再就业优惠证》和小额贷款的审核发放。搞好就业和再就业培训，提高劳动者素质，把人口压力转化为人力资源优势。统筹解决大中专毕业生、复转军人、“4050”人员、城镇新成长劳动力和农村富余劳动力就业问题。公益性岗位优先安排下岗失业人员。保护好民工的合法权益，支持律师事务所及其他社会中介机构从事民工维权活动，切实解决好劳动安全、职业病防护保障差和拖欠民工工资等突出问题。

（二）建立健全社会保障体系。继续巩固“两个确保”100%的成果，做好“三条保障线”的衔接。进一步做好城市居民最低生活保障工作，把符合条件的城市困难居民和企业困难职工纳入低保范围，并逐步合理提高低保标准；继续做好养老、失业、医疗保险制度改革工作，逐步建立与我市经济发展水平相适应的社会保障体系。扩大社会保险覆盖面，抓好个体私营等非公有制企业和灵活就业人员的参保工作，依法把应保未保的企业职工逐步纳入社会保险。抓好社会保险金的清欠、扩面、征缴工作，提高支撑能力。完善社会救助制度，做好城市生活无着的流浪乞讨人员救助管理工作，积极帮助城市特困家庭解决就医看病、子女上学、冬季取暖等方面的实际困难，切实安排好灾区、贫困地区和困难企业群众的生产生活，确保他们有饭吃、有衣穿、有房住。

（三）努力改善人民生活。千方百计增加城乡居民特别是中低收入者的收入，努力提高人民群众的消费能力。结合纠正部门和行业不正之风，进一步清理和取消汽车、住房、教育、医疗等领域的不合理收费。认真落实经济适用住房的优惠政策。严格实行城市教育收费公示制、农村中小学“一费制”、民办学校生均教育成本收费制和高中“择校生”“三限”政策。全面推行县级以上医疗机构药品集中招标采购制度，实行药品和医疗服务价格公示制。以抓好食品、药品“放心工程”为重点，深入开展整顿和规范市场经济秩序工作，依法严厉打击制假、售假和商业欺诈行为。拓宽消费品进入农村市场的渠道，为扩大农村消费创造条件。

八、统筹兼顾，促进经济社会协调发展

（一）促进区域经济加快发展。壮大中心城区规模，发挥中心城区的资金、信息、物资、技术、人才聚集带动效应，培育和发展产业集群，逐步提高产业集中度和专业化协作水平，增强中心城区的辐射功能。进一步支持安顺开发区、西秀新区加快发展，着重引进和发展科技型企业，在以信息化带动工业化的进程中率先实现突破。制定能够发挥各县区比较优势的区域发展规划，根据各地资源特点，优化电力、煤炭、冶金、化工等产业的区域布局，有效配置区域生产力。落实培育支柱财源、扶持县级财政收入上台阶等政策措施，完善激励机制和政策支持体系，合理调整事权，增强县级统筹能力，增强县域经济发展活力。加强乡镇基础设施建设，鼓励多渠道、多形式投资小城镇基础设施建设和兴办公用事业，完善小城镇功能。把发展乡镇企业和小城镇建设有机结合起来，引导乡镇企业进一步向小城镇集中，使乡镇企业成为小城镇的支柱产业。加快户籍管理制度改革，合理引导农村人口向小城镇集中，促进城镇化水平的提高。采取有效措施加强对困难地区的扶持，落实财政转移支付制度，完善县对乡的财政转移支付制度，在确保困难地区机构正常运转的前提下，多方筹措资金，推进贫困地区全面建设。

（二）推进各项社会事业协调发展。继续做好“两基”攻坚工作，全力支持镇宁自治县、关岭自治县通过省“两基”验收，全力支持平坝县通过省“两基”复查。整合教育资源，进一步扩大高中办学规模。积极办好高等教育、民族教育、职业技术教育和成人教育，发展民办教育，重点培养急需的技工人才和专门人才。积极推进科技创新和技术进步，促进科技资源优化组合，加速运用高新技术和先进适用技术改造提升地方特色产业和传统产业。建立健全知识产权制度，逐步在重点产业建立知识产权预警机制。高度重视非典防治工作，以疾病预防控制体系、突发公共卫生医疗救治体系、重大疫情信息网络体系、卫生执法监督体系建设为重点，全面加强公共卫生体系建设。积极探索经营性文化资产管理模式，鼓励社会资本进入文化产业，搞好文物古迹的恢复和保护。把发展体育产业与开展全民健身运动结合起来，鼓励和支持企事业单位和个人兴建适合大众健身需要的体育场所。大力实施人才强市战略，加强公务员人才队伍建设、加强企业经营管理人才队伍建设、加强专业人才队伍建设，积极推进人才资源整体开发，建立和完善人才市场体系，促进人才合理流动，加大引进各类高层次人才和紧缺人才的力度，健全科学的人才评价机制、公平的选拔使用机制和规范有效的人才激励机制，努力形成人尽其才的良好环境。普及科学文化知识，提高全市人民科学文化素质，建设学习型城市。落实民族政策和宗教信仰自由政策，依法管理民族事务和宗教事务。认真实施妇女儿童发展纲要，支持老龄、残疾人事业发展，促进外事、侨务、档案、史志、人防、国防教育等事业全面发展。

（三）搞好人口资源环境保护工作。进一步强化人口与计划生育工作责任制，切实推行人口与计划生育综合改革工作。不断完善“村为主”和“村民自治”的政策措施，确保全市人口出生率和人口自然增长率分别控制在16.5‰、10‰以内，计生率达到90%以上，全面实现“村为主”，“村民自治”的村达到60%。严格按规划有步骤地开发和利用各种自然资源，严格基本建设程序管理，切实防止对资源的浪费、破坏和无序开发。依法整治无证开采、乱采滥挖、采富弃贫、破坏资源等行为。加强国土资源管理，严格执行经营性土地使用权招标拍卖挂牌出让制度。严格依法执行环保标准，逐步推进污染治理市场化。加强酸雨控制区和重点流域的综合整治，搞好水资源的保护利用，开展饮用水源地周边污染源的集中治理，搞好贯城河的污染治理，建设医疗废物处置中心，积极推进城区公交车柴油改汽油的工作，提高城市空气质量。切实增强全社会的节约意识，突出抓好节约能源、节约原材料和节约用水工作，建设节约型社会。

九、加强政治文明和精神文明建设，全力维护社会稳定

（一）积极推进社会主义政治文明。自觉接受市人大的工作监督和法律监督，自觉接受市政协的民主监督，认真办理人大代表的建议、批评和意见，认真办理政协委员的提案。认真听取各民主党派、工商联和无党派人士对政府工作的意见和建议，支持工会、共青团、妇联等人民团体依法按章履行职责，开展工作，不断完善反映社情民意的机制。进一步加大政务公开力度，增强政策法规的透明度，扩大人民群众对行政事务的知情权、参与权和监督权。继续推行村务、厂务公开。搞好基层组织建设，加强对村（居）委会建设的指导。继续抓好“四五”普法工作。全面开展“群众评议机关”活动，接受人民群众的评判和监督。

（二）大力发展社会主义先进文化。把思想道德建设作为精神文明建设的重要任务，继续贯彻落实《公民道德建设实施纲要》，大力倡导和进行社会公德、职业道德、家庭美德建设。培育和弘扬“爱国爱乡，团结和睦，坚韧顽强，负重奋进”的黔中文化精神。加强基层文化设施建设，抓好6个一类扶贫重点乡镇精神文明活动中心和村文化活动室的建设，认真实施第二期“百县千乡宣传文化工程”。继续以“卫生与秩序”、文明城镇竞赛活动等为载体，深入开展文明城市、文明行业、文明单位、文明村镇和贵黄文明大道创建活动。继续开展文化、科技、卫生“三下乡”活动，抓好文化、科技、卫生、法律四进社区服务。“改陋习、树新风”，在全社会努力形成崇尚科学文明的良好风尚。大力提倡移风易俗，加大殡葬改革力度。坚持经济建设与国防建设协调发展，扎实推进国防后备力量建设，深入开展“双拥”模范城（县）创建工作。

（三）切实维护社会稳定。贯彻落实《安全生产法》，严格执行安全生产责任制和安全事故责任追究制。继续深入开展以煤矿、交通、消防为重点的安全生产专项整治。进一步加强煤矿安全生产工作，严防非法无证煤矿死灰复燃，严格实行煤矿管理、技术人员和特殊工种人员持证上岗制度，切实加强国债安全技改资金和煤炭企业维简费的监督管理，确保安全投入。完善公路事故多发地段的安全防护设施，加大对超限运输的整治力度。加强消防基础设施建设。加大对安全事故的查处力度，着力扭转安全生产事故高发的势头，力争安全事故起数和死亡人数有所下降。坚持“打防结合、预防为主”的方针，坚决打击各类刑事犯罪和经济犯罪特别是黑恶势力，坚决扫除“黄、赌、毒”等社会丑恶现象，进一步加强社会治安综合治理；搞好信访工作，依法维护信访群众的合法权益，妥善化解和处置各种矛盾纠纷。高度警惕和防范敌对势力的破坏活动，严厉打击“法轮功”等邪教组织，坚决维护社会稳定。

十、加强政府自身建设，推进政府工作创新

以政府机构改革为契机，进一步推进政府管理创新和职能转变。按照完善社会主义市场经济体制和推进政治体制改革的要求，调整和完善政府机构设置，科学规范政府部门职能，合理划分和界定部门职责和事权，改进管理方式，提高行政效率，降低行政成本，逐步形成行为规范、运转协调、公正透明、廉洁高效的行政管理体制。

努力建设廉洁型政府。全面贯彻落实中纪委三次全会、国务院第二次廉政工作会议和省纪委三次全会精神，按照为民、务实、清廉的要求，着力解决损害群众利益的突出问题，全面推进党风廉政建设和反腐败工作。严格执行党风廉政建设各项规定，全面落实反腐败斗争三项工作，从源头上治理腐败。严格执行建设工程招投标、产权交易等制度。加快推进中介机构与行政主管部门脱钩改制。提倡勤俭办事业，反对铺张浪费，把有限的财力用到群众直接受惠受益的事业上。不搞脱离实际、劳民伤财的“政绩工程”、“形象工程”。

努力建设法治型政府。坚持依法治市，强化法制观念，大力推进社会法治化。认真抓好《行政许可法》的宣传、培训及相关清理工作，切实提高政府的行政效能和公信力。进一步理顺行政执法体制，清理行政执法主体，规范行政执法行为，强化行政执法监督管理，全面推行行政执法责任制和行政执法错案责任追究制。进一步完善工作推进机制，把科学民主决策作为一项基本的工作制度，建立和完善重大问题集体决策、专家咨询、社会公示和社会听证制度以及决策责任制度。认真听取专家对经济社会发展重大决策的意见，特别是对与人民群众切身利益密切相关的决策事项，一定要提高决策的透明度和公众参与度。

努力建设服务型政府。树立以人为本的政府管理思想，着力在提供公共服务、加强政策引导、提高行政效率、优化发展环境上下功夫。在继续加强经济调节、市场监管职能的同时，更加重视政府的社会管理和公共服务职能。进一步解决政府“越位”、“缺位”和“错位”等问题。进一步转变市场监管方式，努力为市场主体提供优质服务和创造良好发展环境。加快电子政务建设，继续办好安顺市人民政府网站、“黄果树之窗”网站和“市长信箱”。

努力建设责任型政府。树立导向正确、全面发展、经得起人民评判和历史检验的政绩观。紧扣发展主题，抓住热点难点问题，一个一个地研究探索，一个一个地突破解决，把关系安顺经济社会发展的事情办好，保证政府工作符合人民群众的根本利益和普遍意愿。针对“行风评议”、“群众评议机关”活动中人民群众提出的意见和建议，认真抓好整改落实。健全调查研究制度，大力精简会议和文件，改进会风和文风，力戒形式主义和官僚主义。建立健全责任考评机制，把敢负责、抓落实作为政治品质的集中体现，把求实效、创业绩作为能力才干的鉴别标准，激励全市公务员在各自岗位上尽心尽力尽责。

努力建设学习型政府。紧密结合我市改革开放的实际，大力倡导学习之风，牢固确立“学习为本，终身学习，学用结合，提高学习力”的理念，深入学习邓小平理论和“三个代表”重要思想，深入学习党的十六大和十六届三中全会精神，认真学习经济、法律、金融、科技和管理等方面的新知识，不断提高政治素质、理论修养和业务水平。在学习中解放思想，在解放思想中凝聚力量，敢于突破不合时宜的观念、做法和体制束缚，始终保持不甘落后、奋发进取的精神状态，开阔视野，谋划全局，不断增强统筹经济社会发展的能力。

各位代表：做好今年的各项工作，任务艰巨，意义重大。让我们在以胡锦涛同志为总书记的党中央领导下，以邓小平理论和“三个代表”重要思想为指导，全面贯彻党的十六大、十六届三中全会、省委九届四次全会和市委一届七次全会精神，按照省委、省人民政府的总体部署，在市委的统一领导下，在市人大、市政协的监督和支持下，凝聚各族各界群众的智慧和力量，抢抓机遇，加快发展，同心同德，扎实工作，努力完成今年经济社会发展各项目标任务，为加快推进安顺小康建设和富民兴安步伐而努力奋斗！
